# Metformin Alleviates Autistic-Like Behaviors Elicited by High-Fat Diet Consumption and Modulates the Crosstalk Between Serotonin and Gut Microbiota in Mice

**DOI:** 10.1155/2022/6711160

**Published:** 2022-02-17

**Authors:** Wenlin Deng, Haoran Ke, Siqi Wang, Zitong Li, Sitao Li, Pinjing Lv, Fang Li, Ye Chen

**Affiliations:** ^1^Department of Gastroenterology, State Key Laboratory of Organ Failure Research, Nanfang Hospital, Southern Medical University, 510515 Guangzhou, Guangdong, China; ^2^Department of Pediatrics, The Sixth Affiliated Hospital, Sun Yat-sen University, 510655 Guangzhou, China; ^3^Department of Gastroenterology, Gastroenterology Endoscopy Center, Hainan General Hospital, Hainan Affiliated Hospital of Hainan Medical University, Haikou 570311, China

## Abstract

The biological mechanisms linking diet-related obesity and autistic behaviors remain unclear. Metformin has proven to be beneficial in the treatment of many syndromes, including autism spectrum disorder. Therefore, the aim of this study was to assess whether metformin treatment could ameliorate metabolic and behavioral alterations in C57BL/6 mice kept on a high-fat diet (HFD), and whether these changes were related to modifications in the gut microbiota and 5-HT levels. As expected, ten weeks of HFD ingestion increased body weight, adiposity, and glucose levels. HFD-fed mice showed a marked aggravation of repetitive behaviors (marble burying and self-grooming), and this was prevented by metformin administration. In addition, HFD-fed mice increased the total distance travelled in the open field test. This hyperactivity was counteracted by metformin cotreatment. In the elevated plus maze test, HFD-fed mice showed a reduced number of entries into the open arms. Interestingly, both HFD and metformin cotreatment increased social interactions in the three-chamber test. HFD increased the levels of intestinal tryptophan and 5-hydroxyindoleacetic acid. Metformin stimulated gut tryptophan and promoted the synthesis of 5-HT in the HFD group. *Lactococcus*, *Trichococcus*, *Romboutsia*, and *Faecalibaculum* were enriched in HFD-fed mice, whereas the HFD group cotreated with metformin was enriched in *Intestinimonas* and *L. reuteri*. *Faecalibacterium* was positively correlated with sociability and 5-HT pathway components in mice that received metformin. In summary, HFD consumption elicited a complex phenotype comprising higher levels of anxiety-like and repetitive behaviors but also increased sociability. Metformin could potentially improve HFD-induced disorders in the autistic spectrum through a mechanism involving positive modulation of 5-HT levels in the gut and its microbiota composition.

## 1. Introduction

In recent years, the steadily increasing rates of obesity have presented a major challenge to healthcare systems worldwide [1]. The association between chronic consumption of a high-fat diet (HFD) and the development of obesity and its metabolic comorbidities is well established [2]. There is also an emerging body of evidence linking HFD and psychopathology in both humans and animals, including anxiety and depressive-like behaviors [3, 4]. However, few of these studies have focused on social behaviors related to the autism spectrum disorder (ASD). ASD is characterized by two core symptom domains: abnormal social communication and behaviors, and repetitive behaviors [5]. In recent years, as obesity rates have risen, there has been a similar surge in the number of diagnosed ASD cases. A recent study has shown that chronic HFD ingestion diminishes the preference for social novelty in mice [6].

HFD consumption can rapidly alter the composition of the gut microbiota in mice [7]. The metabolic effects of HFD consumption on the gut may influence brain development via the so-called “gut-brain axis” [8]. Studies have shown that alterations in the gut microbiota and disruptions in the function of the intestinal barrier are present in mouse models of autism, suggesting that the gut microbiota may play a crucial role in ASD development [9]. However, the relationship between HFD consumption, the composition of the intestinal microbiota, and the display of social behaviors remain unclear.

At present, no drugs are available to treat the core symptoms of ASD. Metformin (Met) is the most commonly used drug for treating type 2 diabetes, but it has been shown to have many additional physiological effects [10]. Chronic oral metformin administration partially prevents the development of HFD-induced cognitive deficits in animals and humans [11]. Met has also been shown to alleviate anxiety-like behavior induced by a HFD and to ameliorate some abnormal behaviors in a mouse model of autism [12, 13]. We have recently described a protective effect of metformin against mucus barrier dysfunction via the gut microbiota in mice with dextran sulfate sodium-induced colitis [14].

In this study, we aimed to determine whether HFD consumption exacerbates anxiety and autistic-like behaviors in C57BL/6 mice and whether this can be ameliorated by metformin administration. We also focused on the serotonergic (5-hydroxytryptamine, 5-HT) system as a potential mediator in the crosstalk between the gut and the brain, since more than 90% of the 5-HT in the body is synthesized by enterochromaffin cells (ECs) located in the gastrointestinal (GI) tract [15].

## 2. Materials and Methods

### 2.1. Animals and Metformin Supplementation

Four-week-old male C57BL/6 mice were purchased from the Southern Medical University (SMU, Guangzhou, China). The mice were housed in a room with controlled temperature (22°C) and a 12-hour light/dark cycle with free access to water and food. All animals were fed custom-made HFD based on Research Diets D12451 (Specialty Feeds, Glen Forrest, WA, USA) for eight consecutive weeks. Metformin (400 mg/kg) was administered intragastrically during two weeks based on previous research and our own findings [14]. Mice were sacrificed after eight weeks of dietary treatment and one week after behavioral testing. All experiments involving animals were approved by the Institutional Animal Care and Use Committee of the SMU.

### 2.2. Behavioral Tests

#### 2.2.1. Marble Burying Test

Changes in repetitive behavior were evaluated via the marble burying test in line with previously described methods [16]. Each mouse was placed in a plastic container (46 cm long × 24 cm wide × 21 cm deep) filled with clean woodchip bedding to a height of 5 cm. Twenty glass marbles (1.5 cm in diameter), arranged in five rows of four marbles, were placed on top of the bedding. Mice were allowed to bury marbles freely for 30 min. At the end of the test, the number of buried marbles was recorded, with marbles covered in at least two-thirds of their surface by bedding considered as buried. After completion of each test, the marbles were thoroughly cleaned, and new bedding was provided for the testing chamber.

#### 2.2.2. Open Field Task

The open field task (OFT) was used to assess psychomotor outcomes and exploratory behavior in the experimental animals. As previously described [17], each individual animal was placed in the central zone of the OFT arena, and video was recorded for 20 min using a camera mounted above the arena. The time spent in the central zone and the total distance traveled were analyzed for each mouse using a VideoMot2 tracking system (TSE systems, Germany). The arena was cleaned with a 70% alcohol solution between two consecutive tests, and a 5–7 min interval between cleaning and the start of the next testing session was observed to allow for odor dissipation.

#### 2.2.3. Elevated Plus Maze

The elevated plus maze (EPM) test was performed similarly to the OFT. Briefly, mice were placed in a four-arm plus maze. Each arm was 40 cm long and 10 cm wide, and the walls were 40 cm high. The central area of the maze (10 cm × 10 cm) was not considered to be either a closed or open space [18]. Each mouse was initially placed in the central area with the snout pointing at one of the open arms. Each individual test lasted 5 min, and the maze was cleaned with a 70% alcohol solution between consecutive tests.

#### 2.2.4. Three-Chamber Test

The three-chamber test was used to measure sociability as described previously [18]. In brief, the three-chambered apparatus consisted of a Plexiglas box divided into three interconnected chambers with small, retractable entryways. After 5 min of habituation, a mouse was allowed to explore the setup and interact either with an empty wire cup placed in one of the chambers or with a similar wire cup containing an unfamiliar conspecific in the opposite chamber. The conspecific was matched to the experimental subject by sex, age, and strain, representing sociability and social novelty recognition. During the first phase of testing, the subjects were allowed to choose between two identical nonsocial stimuli, and during the second phase, the choices offered were a nonsocial stimulus and a social stimulus. The time spent interacting with each cup and the total distance traversed were measured and analyzed using the TSE tracking system. After each test, the chamber was cleaned with a 70% alcohol solution. Time spent in the chamber with the social stimulus was considered as an estimate of social approach behavior.

#### 2.2.5. Self-Grooming

The mice were scored for spontaneous grooming behavior, as described earlier [18]. Each mouse was placed individually in a three-chamber test arena and scored for ten minutes after completion of the previous test. The pattern of grooming in rodents usually proceeds in a cephalocaudal direction. The testing arena was cleaned with water and a 70% ethanol solution after each consecutive test.

### 2.3. Oral Glucose Tolerance Test

To investigate the response to glucose, an oral glucose tolerance test (OGTT) was performed after 6 hours of fasting. A 20% glucose solution was administered by gavage (1 g/kg body weight). Blood samples were obtained via tail snip at 0, 15, 30, 60, or 90 min after administration, and fasting glucose levels were measured using a glucometer (One Touch Ultra, Johnson and Johnson, New Brunswick, NJ, USA) [19].

### 2.4. Quantitative Real-Time PCR (qRT-PCR) Analysis

Total RNA was extracted from the distal colon, and qRT-PCR was performed using SYBR® Premix Ex Taq™ (Takara, Beijing, China) in a Roche LightCycler® 480II equipment (Roche Diagnostics, Rotkreuz, Switzerland). The proteins coded by the genes amplified and their corresponding primer sequences were as follows: serotonin reuptake transporter (SERT) (forward, 5′-GCT CAT CTT CAC CATTAT CTA CTT C-3′; reverse, 5′-AGT TTC TGCCAG TTG GGT TTC-3′) and tryptophan hydroxylase 1 (TPH1) (forward, 5′-CCT GCA AAC AGG AAT GTCT-3′; reverse, 5′-TCT GGA CTG ATG CTC AAA GG-3′). Data were analyzed using the comparative cycle threshold method (2^−*ΔΔ*Ct^), with *β*-actin as the reference gene.

### 2.5. High Performance Liquid Chromatography (HPLC) Analysis

Mice were sacrificed by decollation. The colons were weighed and then homogenized in a solution of acetonitrile with 0.2% formic acid in a 1 : 10 *w*/*v* proportion using an ultrasonic homogenizer. After centrifugation at 12000 rpm at 4°C for 20 min, the supernatant was collected, filtered through a 0.22 *μ*m syringe filter, and stored at −80°C until HPLC analysis. The concentrations of the 5-HT metabolic system components were measured using the Prelude SPLCTM system in a TSQ Endura triple-stage quadrupole mass spectrometer (Thermo Fisher Scientific, Waltham, MA, USA). The chromatographic column was a HSST3 2.1 × 100 mm, 1.7 *μ*m (Waters Corporation, Milford, MA, USA). The mobile phases consisted of (A) 0.4% formic acid, 1 mM ammonium formate dissolved in water, and (B) 0.1% formic acid dissolved in methyl alcohol.

### 2.6. Microbiome Analysis

The 16S rRNA gene sequencing procedure was performed by the Novogene Institute (Beijing, China). Briefly, total genomic DNA was extracted using the CTAB/SDS method. DNA concentration and purity were monitored using 1% agarose gels. Amplicon generation PCR was carried out in a 30 *μ*L reaction volume with 15 *μ*L of Phusion® High-Fidelity PCR Master Mix (New England Biolabs, Ipswich, MA, USA), 0.2 *μ*M of forward and reverse primers, and approximately 10 ng of template DNA. Sequencing libraries were generated using the Ion Plus Fragment Library Kit 48 runs (Thermo Fisher Scientific) following the manufacturer's recommendations. The library quality was assessed on a Qubit® 2.0 Fluorometer (Thermo Fisher Scientific). Finally, the library was sequenced on an Ion S5™ XL platform (Thermo Fisher Scientific), and 400 bp/600 bp single-end reads were generated.

### 2.7. Data Analysis

Data were analyzed using Prism 8 (GraphPad Software, San Diego, CA, USA) and R software (The R Foundation, Vienna, Austria). Results are presented as mean ± standard error of the mean (SEM). Statistical analyses were performed using two-tailed unpaired Student's *t*-tests and two-way ANOVA. The linear relationship was analyzed using Pearson's *r* after a Shapiro–Wilk normality test. *p* values under 0.05 were considered statistically significant, and the following symbols were used to denote levels of significance: ^∗^*p* < 0.05, ^∗∗^*p* < 0.01, and ^∗∗∗^*p* < 0.001.

## 3. Results

### 3.1. Metformin Supplementation Ameliorates Body Weight and Fasting Blood Glucose in HFD-Fed Mice

**Figure 1:**
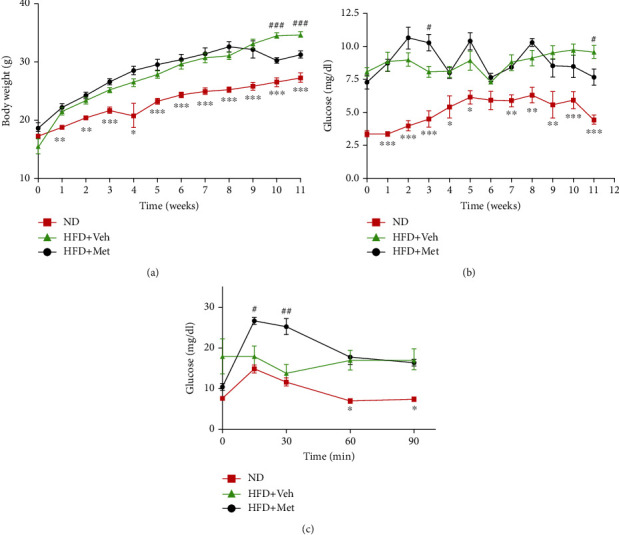
Effect of HFD and metformin treatment on body weight, fasting blood glucose levels, and glucose tolerance. (a) Time curve of body weight gain. (b) Time curve of fasting blood glucose levels. (c) OGTT response in HFD-fed mice after metformin administration (*n* = 5 − 6). Values are presented as mean ± SEM. ^∗^*p* < 0.05, ^∗∗^*p* < 0.01, and ^∗∗∗^*p* < 0.005 superscripts indicate significant differences between the ND vs. HFD + Veh groups; ^#^*p* < 0.05, ^##^*p* < 0.01, and ^###^*p* < 0.001 superscripts indicate significant differences between the HFD + vehicle vs. HFD + Met groups. OGTT: oral glucose tolerance test; ND: normal diet; HFD: high-fat diet; Met: metformin.

To study the effects of metabolic disorders, the C57BL/6 mice were divided into three groups: the first group was fed a normal diet (ND) as a control, while the second group was fed a HFD (HFD + vehicle), and the third group was fed a HFD and cotreated with metformin (HFD + Met) via oral gavage. As previously observed, body weight gain and glucose levels were significantly increased in the HFD + vehicle group compared with those that were fed a ND. Metformin supplementation (400 mg/kg) mitigated the HFD-mediated weight gain and decreased glucose levels (Figures 1(a) and 1(b)). However, metformin treatment did not improve glucose tolerance in HFD-fed mice (Figure 1(c)).

### 3.2. HFD Exacerbates Repetitive Behaviors and Metformin Alleviates Them

**Figure 2:**
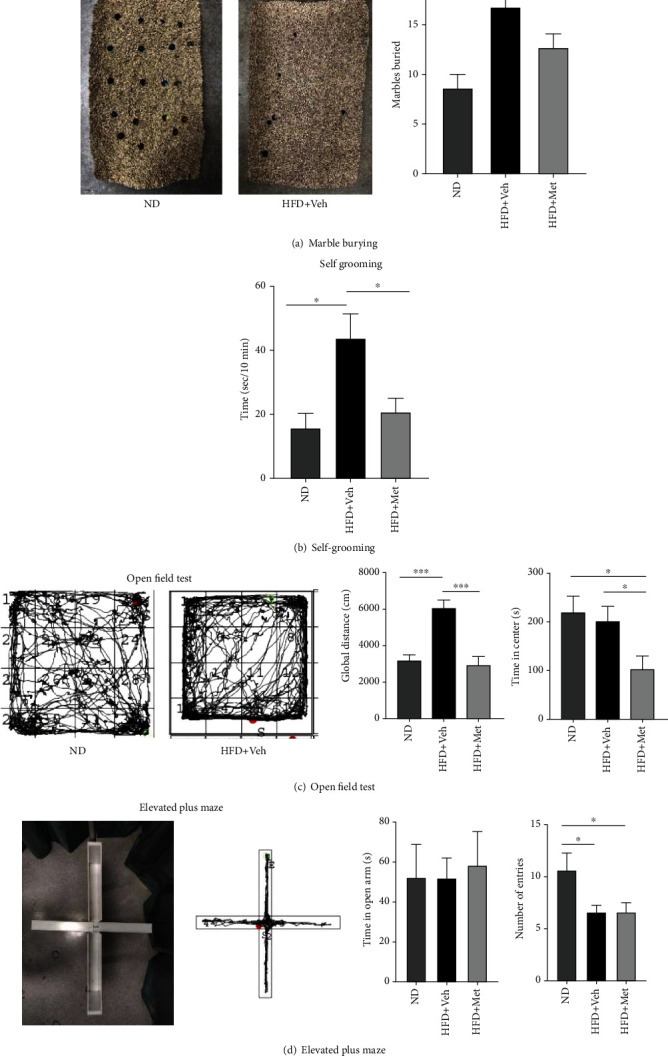
Effects of HFD and metformin on the display of repetitive and stereotyped behaviors in mice. (a, b) HFD-fed mice buried more marbles and displayed more self-grooming compared to those in the ND group, suggesting enhanced repetitive behavior. (c) A HFD increased the total distance traversed in the open field test (OPT) but had no significant effect on time spent on the central area. (d) Mice from the ND entered more times into the open arms in the elevated plus maze (EPM) tests. Data are presented as mean ± SEM (*n* = 8 − 9). ^∗^*p* < 0.05, ^∗∗^*p* < 0.01, and ^∗∗∗^*p* < 0.001 compared with the control group. Veh: vehicle.

Marble burying and self-grooming tests were used to measure repetitive and stereotyped behaviors. The HFD-fed mice displayed higher levels of marble-burying and self-grooming behavior than the ND-fed mice (Figures 2(a) and 2(b), *p* < 0.05). Metformin administration significantly reversed this increase in both tests. Together, these results indicate that HFD aggravates repetitive behaviors, and metformin alleviates them.

### 3.3. Effects of HFD on Open Field and Elevated Plus Maze Tests

The OPT and EPM tests were used to measure activity and anxiety-like behavior in a novel environment. HFD consumption significantly increased the length of the traveled path compared with ND controls in the OPT, and metformin reversed this effect. The HFD + Met group spent less time in the central zone (Figure 2(c), *p* < 0.05). In the EPM, no difference in time spent at the open arms was observed between the HFD and ND groups (Figure 2(d), *p* > 0.05). However, mice from the ND group entered more times into the open arms compared to those from the other groups (Figure 2(d)*p* < 0.05). In summary, these observations suggest that HFD induces anxiety-like behavior, and metformin ameliorates the hyperactivity displayed by HFD-fed mice.

### 3.4. Both HFD and Metformin Increased Sociability in the Three-Chamber Test

**Figure 3:**
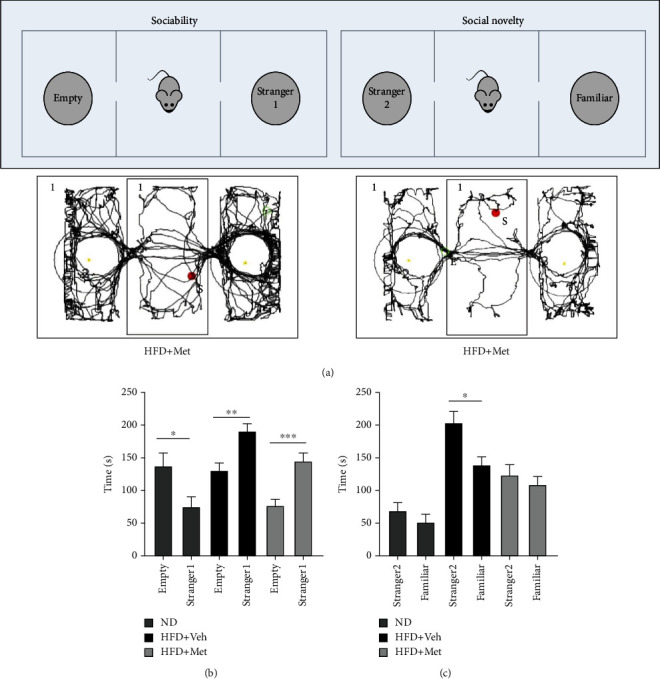
HFD and metformin increased sociability in the three-chamber test. (a) Experimental design for the three-chamber test for sociability/social novelty. (b) Mice from the HFD + vehicle and HBD + metformin groups spent significantly more time in the chamber with the unfamiliar conspecific, suggesting more sociability. (c) Mice from the HFD group showed significant preference for a novel over a familiar conspecific. Data are presented as the mean ± SEM (*n* = 8). ^∗^*p* < 0.05 and ^∗∗^*p* < 0.01 compared with the control group.

The three-chamber test examined sociability and response to social novelty (Figure 3(a)). Mice typically spend more time exploring the chamber containing the unfamiliar conspecific than the empty chamber [20]. Interestingly, mice from the HFD + vehicle and the HFD + Met groups spent more time in the chamber containing the unfamiliar conspecific in the social behavior test, but mice from the ND group did the opposite (Figure 3(b), *p* < 0.05). In the social novelty test, the HFD group showed a significant preference for social novelty (Figure 3(c), *p* < 0.05). Overall, we observed that either HFD + vehicle or HFD + Met treatment elicited a preference for social proximity.

### 3.5. Impact of HFD and Metformin Treatment on the 5-HT Pathway

**Figure 4:**
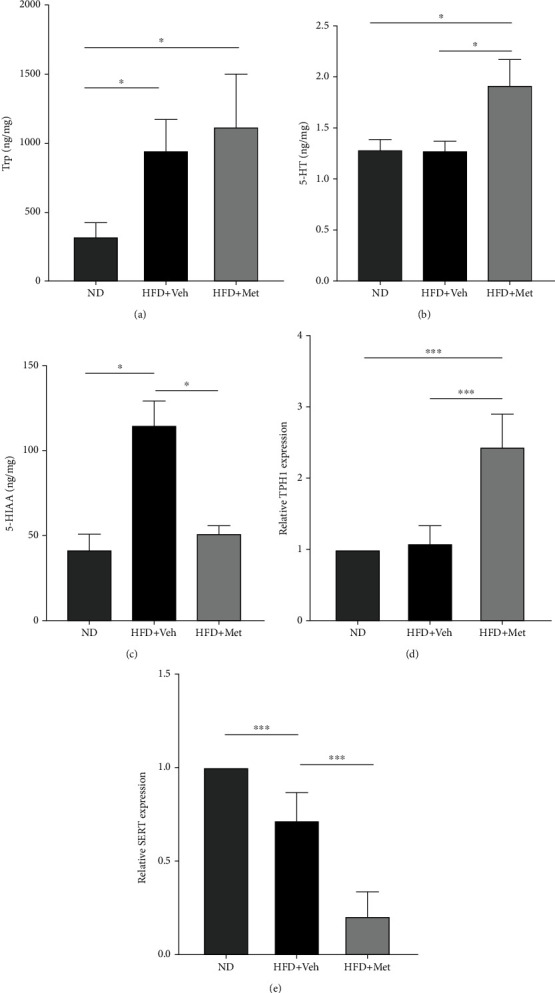
HFD and metformin regulate the 5-HT pathway in the gut.(a–c) HPLC analysis of tryptophan, 5-HT, and the serotonin metabolite 5-HIAA levels in the colon. (d, e) Messenger RNA expression of tryptophan hydroxylase 1 (TPH1) and serotonin reuptake transporter (SERT) in the colon (*n* = 6). Data are presented as mean ± SEM. ^∗^*p* < 0.05. Trp: tryptophan; 5-HT: 5-hydroxytryptamine; 5-HIAA: 5-hydroxyindoleacetic acid.

In the GI tract, ECs produce the majority of 5-HT in the body from dietary tryptophan metabolized by TPH1. 5-Hydroxyindoleacetic acid (5-HIAA) is the main metabolite of 5-HT, which is inactivated by SERT expressed in intestinal epithelial cells [21]. We used HPLC to characterize the 5-HT pathway in the colon, and the results showed that tryptophan levels in the HFD + vehicle and the HFD + Met groups were remarkably increased compared to those in the ND group (Figure 4(a), *p* < 0.05), whereas 5-HT levels were significantly increased in the HFD + Met group (Figure 4(b)*p* < 0.05). The levels of 5-HIAA in the HFD + vehicle group were significantly higher (Figure 4(c), *p* < 0.05). We observed an upregulation of the TPH1 gene expression in the HFD + Met group and a downregulation of the SERT gene expression in the HFD + vehicle and the HFD + Met groups (Figures 4(d) and 4(e), *p* < 0.001). These results suggest that metformin stimulated 5-HT in HFD-fed mice by increasing tryptophan and reducing 5-HT metabolism and that HFD feeding increases intestinal tryptophan and enhances 5-HT metabolism.

### 3.6. HFD Reprogramming of the Gut Microbiota

**Figure 5:**
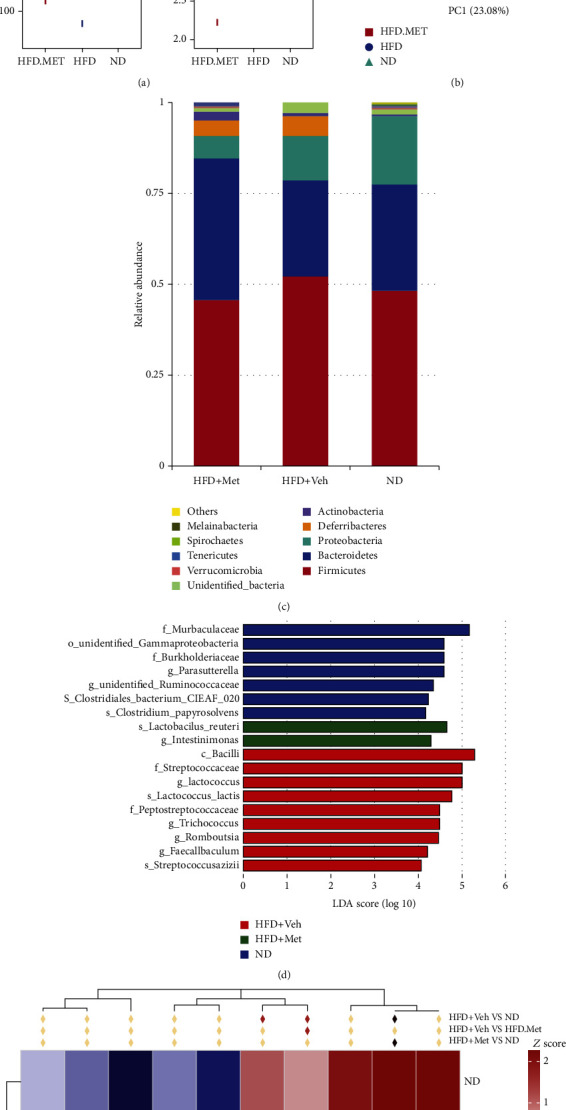
HFD reprograms the gut microbiota. (a) Number of observed OTU index was significantly higher in the ND group. (b) Principal coordinate analysis (PCoA) based on an unweighted UniFrac matrix shows that the overall fecal microbiota composition is different between three groups. (c) Abundance of *Firmicutes* and *Bacteroidetes*. The HFD group shows an increased F/B ratio. (d) Linear discriminant analysis effect size (LEfSe) method results on mouse gut microbiomes (LDA threshold of 4). (e) Heat map of the top 10 phyla in the three groups among three groups (*n* = 4 − 5).

Compared to the ND group, HFD significantly decreased the richness of the gut microbiota, and metformin treatment failed to reverse this effect (Figure 5(a)). However, the diversity shown by the Simpson index was not significantly different. Principal coordinate analysis (PCOA) results demonstrate the effects of different diets on microbial *β* diversity (Figure 5(b)). The microbial communities exhibited distinct clusters in mice fed with HFD alone and with HFD supplemented with metformin. The microbial composition in the mice from the HFD + Met group was more similar to that observed in mice from the HFD + vehicle group than to that from the mice that were fed a normal diet. The ratio of *Firmicutes* to *Bacteroidetes* increased in the HFD + vehicle group, and metformin increased *Bacteroidetes* abundance (Figure 5(c)). The linear discriminant analysis effect size (LEfSe) method was used to determine the taxa at different taxonomic levels that were enriched between the groups (Figure 5(d)). At the genus level, compared to the ND group, the HFD group showed a relative abundance of *Lactococcus*, *Trichococcus*, *Romboutsia*, and *Faecalibaculum*. *Intestinimonas* and *Lactobacillus reuteri* were enriched in the HFD + Met group (LDA ≥ 4). In addition, compared with the ND group, the relative abundance of *Melainabacteria* in the HFD + vehicle and HFD + Met groups was remarkably decreased (*p* < 0.01). However, compared to the HFD + vehicle group, the relative abundance of *Tenericutes* in the HFD + Met was overrepresented (Figure 5(e), *p* < 0.05).

**Figure 6:**
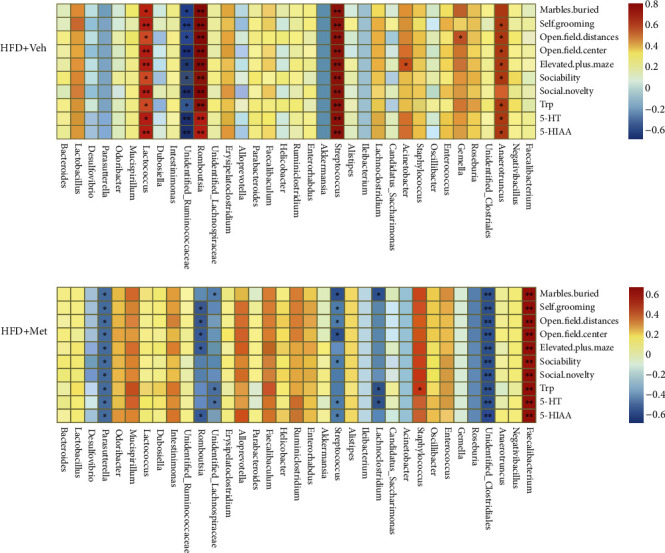
Correlation matrix showing positive (red) and negative (blue) associations between behavioral scores, levels of the 5-HT pathway components, and relative abundance of bacterial genera.

Next, we analyzed the association between the top 35 relative abundances of bacteria, the behavioral phenotype, and 5-HT products and found that *Lactococcus*, *Streptococcus*, and *Romboutsia* were strongly positively associated with behavioral scores and 5-HT products in the HFD + vehicle group (Figure 6), whereas *Faecalibacterium* was positively correlated in the HFD + Met group (Figure 6). *Streptococcus* and *Parasutterella* were negatively correlated with sociability in the HFD + Met group.

## 4. Discussion

Our research was aimed at exploring the impact of a high-fat diet on the display of autistic-like symptoms in C57BL/6 mice and the effect of metformin on these animals. As expected, mice that consumed a HFD displayed significantly increased body weight gain and fasting glucose levels. Metformin supplementation effectively prevented this high fasting glucose levels and weight gain triggered by the HFD. When the behaviors were analyzed, we found that a HFD exacerbated the anxiety-like phenotype in a way that was consistent with previous reports. Notably, metformin supplementation suppressed the repetitive behaviors and hyperactivity observed in mice fed with a HFD but did not have an antidepressive effect. More importantly, the HFD and HFD plus metformin treatment indicated a rescue in sociability as measured in the three-chamber test.

This result is consistent with the findings that HFD ingestion or obesity elicits anxiogenic/depressive-like symptoms [2, 3, 22, 23]. Numerous studies have shown that HFD induces anxiety-like behavior in mice, and interestingly, HFD withdrawal reverses it [24]. However, there has been less focus on sociability. In agreement with previous reports on social behaviors, one study revealed that a HFD improves social interaction in C57BL/6 mice [25]. Another study reported that mice fed with a high-fat and high-sugar (HFHS) diet exhibited impaired social memory, but no deficits in sociability [26]. On the other hand, Hassan and colleagues reported that a HFD led to a depression-like phenotype evidenced by reduced sociability [27]. Furthermore, a study reported that three weeks of co-treatment with metformin alleviated anxiety-like behavior in mice on a HFD [28]. In our study, metformin administration failed to exert any therapeutic effect. These inconsistent results may be due to potentially insufficient duration of treatment in our protocol or to the fact that HFD-induced anxiety and depression-like behaviors may not share a similar pathophysiology with sociability deficits.

We hypothesized that consumption of a HFD resulted in gut dysbiosis and was also connected with 5-HT levels, which could in turn be linked to autistic and anxiety-like behaviors. Researchers have shown that male germ-free mice have significantly higher levels of plasma tryptophan but considerably lower levels of plasma serotonin than typically colonized mice, implying that the absence of gut microbes affects the peripheral conversion of tryptophan to serotonin [29]. We used HPLC to characterize the 5-HT pathway in the gut. It is well established that 5-HT levels are closely related to anxiety and depression. Selective 5-HT reuptake inhibitors are the most commonly prescribed antidepressants and antianxiety medications. Previous findings showed that HFD feeding significantly increased plasma and intestinal 5-HT levels [30]. Our results indicate that a HFD stimulates tryptophan levels in the gut. However, 5-HT did not significantly increase in HFD-fed mice, which may be due to enhanced metabolism (Figure 4(c)) The levels of 5-HT, on the other hand, were significantly increased in HFD mice that received metformin.

Previous studies revealed that metformin has several effects within the gut, including 5-HT regulation [31]. Zemdegs and colleagues found that HFD induced depressive-like symptoms associated with decreased extracellular 5-HT levels in the hippocampus, which may result from increased sensitivity of the dorsal raphe 5-HT1A autoreceptor [23]. However, in our study, metformin improved sociability but did not ameliorate anxiety-like behaviors.

Since social behavior deficits are the core symptoms of autism that are most resistant to treatment, the effect of 5-HT levels on sociability impairment remains unclear. In the present study, mice treated with HFD plus metformin displayed greater sociability as well as an increase in 5-HT levels. There is mounting evidence that the serotonergic system is implicated in social cognition and social interaction [32]. Walsh and colleagues reported that 5-HT release in the nucleus accumbens rescues social deficits in a mouse model of autism [33]. A recent report indicated that systemic enhancement of serotonin signaling reverses social deficits in several mouse models of ASD [34]. Tryptophan depletion has been shown to reduce the preference for social interaction in C57BL/6 and BTBR mice, while 5-HT levels were at the same time decreased [35], and these changes matched the clinical responses observed in autistic patients. In contrast, prenatal metformin exposure or genetic ablation of the organic cation transporter 3 suppresses the preference for social interaction in male mice, which is associated to serotonin metabolism [36]. In preclinical studies, activation of the 5-HT7 receptor corrects deficits in mouse models of Fragile X and Rett syndromes, which are the leading monogenic causes of ASD. These inconsistent findings suggest that the balance of 5-HT levels may play a role in sociability behaviors [37].

The effects of a HFD on the gut microbiome have been extensively studied in rodents, and the altered abundance of the *Bacteroidetes* and *Firmicutes* phyla (F/B ratio) has been described as typical [38, 39]. In agreement with previous studies, our findings show that HFD intake increased the F/B ratio. However, metformin cotreatment did not improve the balance of the intestinal flora. A previous report showed that metformin alters the microbiome in both mice and humans and, in contrast with the effect of a HFD, causes an overall decrease in the bacterial diversity of the mouse microbiome [40, 41]. The gut microbiota has been widely investigated for its role in anxiety and ASD. Increasing evidence suggests that the gut microbiome affects 5-HT levels as well as behavior. For example, some bacterial species from the *Enterococcus*, *Escherichia*, and *Streptococcus* genuses are capable of secreting serotonin directly [42], and *Bacteroides fragilis* ameliorated some autistic-like phenotypes in mice. A study indicated that probiotics, including *Lactococcus*, reduce cognitive reactivity to the negative mood via alterations in the activity of brain regions that control the central processing of emotions and sensations [43]. In the present study, we also found that the genus *Lactococcus* was relatively abundant in the HFD-fed group, and *L. reuteri* was abundant in the HFD + Met group. In particular, *L. reuteri* has been shown to selectively rescue social deficits in genetic, environmental, and idiopathic ASD models via the vagus nerve [44, 45]. Interestingly, in our study, HFD and metformin treatment ameliorated social interaction, which may be related to the relative abundance of these microbes. *Lactococcus* was also strongly positively associated with behavioral scores and the 5-HT system in the HFD group. Furthermore, *Tenericutes* abundance in the HFD + Met group was significantly higher than in the HFD + vehicle group. Bacteria from the *Tenericutes* phylum have been found to be positively associated with modulation of the immune system and have beneficial effects on intestinal integrity [46]. However, the precise relationship between gut microbiota and anxiety-like and autistic behaviors remains obscure.

## 5. Conclusion

In summary, the present study demonstrated that a HFD enhanced the display of anxiety-like, repetitive behaviors and ameliorated social interaction in male mice. Metformin could serve as a potential therapeutic agent against ASD-related behavior induced by chronic HFD ingestion, and this may be related to the 5-HT levels in the gut and to its microbiota composition. Although the details warrant further investigation, deciphering the mechanism by which HFD ingestion triggers autistic-like behavior will help to better understand the gut-brain axis in the future.

## Data Availability

The data used to support the findings of this study are available from the corresponding authors upon request.

## References

[1] Bhupathiraju S. N., Hu F. B. (2016). Epidemiology of obesity and diabetes and their cardiovascular complications. *Circulation Research*.

[2] Sweeney P., O'Hara K., Xu Z., Yang Y. (2017). HFD-induced energy states-dependent bidirectional control of anxiety levels in mice. *International Journal of Obesity*.

[3] Rajan T. M., Menon V. (2017). Psychiatric disorders and obesity: a review of association studies. *Journal of Postgraduate Medicine*.

[4] Anderson S. E., Cohen P., Naumova E. N., Must A. (2006). Association of depression and anxiety disorders with weight change in a prospective community-based study of children followed up into adulthood. *Archives of Pediatrics & Adolescent Medicine*.

[5] American Psychiatric Association (2013). *Diagnostic and Statistical Manual of Mental Disorders*.

[6] Zilkha N., Kuperman Y., Kimchi T. (2017). High-fat diet exacerbates cognitive rigidity and social deficiency in the BTBR mouse model of autism. *Neuroscience*.

[7] Foley K. P., Zlitni S., Denou E. (2018). Long term but not short-term exposure to obesity related microbiota promotes host insulin resistance. *Nature Communications*.

[8] Cryan J. F., O'Riordan K. J., Cowan C. S. M. (2019). The microbiota-gut-brain axis. *Physiological Reviews*.

[9] Fattorusso A., Di Genova L., Dell'Isola G. B., Mencaroni E., Esposito S. (2019). Autism spectrum disorders and the gut microbiota. *Nutrients*.

[10] Ma W., Chen J., Meng Y., Yang J., Cui Q., Zhou Y. (2018). Metformin alters gut microbiota of healthy mice: implication for its potential role in gut microbiota homeostasis. *Frontiers in Microbiology*.

[11] Tanokashira D., Kurata E., Fukuokaya W. (2018). Metformin treatment ameliorates diabetes-associated decline in hippocampal neurogenesis and memory via phosphorylation of insulin receptor substrate 1. *FEBS Open Bio*.

[12] Gantois I., Khoutorsky A., Popic J. (2017). Metformin ameliorates core deficits in a mouse model of fragile X syndrome. *Nature Medicine*.

[13] Arika W. M., Kibiti C. M., Njagi J. M., Ngugi M. P. (2019). Effects of DCM leaf extract of Gnidia glauca (Fresen) on locomotor activity, anxiety, and exploration-like behaviors in high-fat diet-induced obese rats. *Behavioural Neurology*.

[14] Deng J., Zeng L., Lai X. (2018). Metformin protects against intestinal barrier dysfunction via AMPK *α*1-dependent inhibition of JNK signalling activation. *Journal of Cellular and Molecular Medicine*.

[15] Mawe G. M., Hoffman J. M. (2013). Serotonin signalling in the gut-functions, dysfunctions and therapeutic targets. *Nature Reviews. Gastroenterology & Hepatology*.

[16] Angoa-Pérez M., Kane M. J., Briggs D. I., Francescutti D. M., Kuhn D. M. (2013). Marble burying and nestlet shredding as tests of repetitive, compulsive-like behaviors in mice. *Journal of Visualized Experiments*.

[17] Peñagarikano O., Abrahams B. S., Herman E. I. (2011). Absence of CNTNAP2 leads to epilepsy, neuronal migration abnormalities, and core autism-related deficits. *Cell*.

[18] Silverman J. L., Babineau B. A., Oliver C. F., Karras M. N., Crawley J. N. (2013). Influence of stimulant-induced hyperactivity on social approach in the BTBR mouse model of autism. *Neuropharmacology*.

[19] Nagy C., Einwallner E. (2018). Study of glucose metabolism in high-fat diet-fed mice using oral glucose tolerance test (OGTT) and insulin tolerance test (ITT). *Journal of Visualized Experiments*.

[20] Aidanovich-Beilin O., Lipina T., Vukobradovic I., Roder J., Woodgett J. R. (2011). Assessment of social interaction behaviors. *JoVE (Journal of Visualized Experiments)*.

[21] Israelyan N., Margolis K. G. (2018). Serotonin as a link between the gut-brain-microbiome axis in autism spectrum disorders. *Pharmacological Research*.

[22] Eudave D. M., BeLow M. N., Flandreau E. I. (2018). Effects of high fat or high sucrose diet on behavioral-response to social defeat stress in mice. *Neurobiology of Stress*.

[23] Zemdegs J., Quesseveur G., Jarriault D., Pénicaud L., Fioramonti X., Guiard B. P. (2016). High-fat diet-induced metabolic disorders impairs 5-HT function and anxiety-like behavior in mice. *British Journal of Pharmacology.*.

[24] Krishna S., Lin Z., de La Serre C. B. (2016). Time-dependent behavioral, neurochemical, and metabolic dysregulation in female C57BL/6 mice caused by chronic high-fat diet intake. *Physiology & Behavior*.

[25] Takase K., Tsuneoka Y., Oda S., Kuroda M., Funato H. (2016). High-fat diet feeding alters olfactory-, social-, and reward-related behaviors of mice independent of obesity. *Obesity*.

[26] Reichelt A. C., Gibson G. D., Abbott K. N., Hare D. J. (2019). A high-fat high-sugar diet in adolescent rats impairs social memory and alters chemical markers characteristic of atypical neuroplasticity and parvalbumin interneuron depletion in the medial prefrontal cortex. *Food & Function*.

[27] Hassan A. M., Mancano G., Kashofer K. (2019). High-fat diet induces depression-like behaviour in mice associated with changes in microbiome, neuropeptide Y, and brain metabolome. *Nutritional Neuroscience*.

[28] Ji S., Wang L., Li L. (2019). Effect of metformin on short-term high-fat diet-induced weight gain and anxiety-like behavior and the gut microbiota. *Frontiers in Endocrinology*.

[29] Clarke G., Grenham S., Scully P. (2013). The microbiome-gut-brain axis during early life regulates the hippocampal serotonergic system in a sex-dependent manner. *Molecular Psychiatry*.

[30] Pan Q., Liu Q., Wan R. (2019). Selective inhibition of intestinal 5-HT improves neurobehavioral abnormalities caused by high-fat diet mice. *Metabolic Brain Disease*.

[31] Banskota S., Ghia J. E., Khan W. I. (2019). Serotonin in the gut: blessing or a curse. *Biochimie*.

[32] Sarkar A., Harty S., Johnson K. V. (2020). The role of the microbiome in the neurobiology of social behaviour. *Biological Reviews*.

[33] Walsh J. J., Christoffel D. J., Heifets B. D. (2018). 5-HT release in nucleus accumbens rescues social deficits in mouse autism model. *Nature*.

[34] Walsh J. J., Llorach P., Cardozo Pinto D. F. (2021). Systemic enhancement of serotonin signaling reverses social deficits in multiple mouse models for ASD. *Neuropsychopharmacology*.

[35] Zhang W. Q., Smolik C. M., Barba-Escobedo P. A. (2015). Acute dietary tryptophan manipulation differentially alters social behavior, brain serotonin and plasma corticosterone in three inbred mouse strains. *Neuropharmacology*.

[36] Garbarino V. R., Santos T. A., Nelson A. R. (2019). Prenatal metformin exposure or organic cation transporter 3 knock-out curbs social interaction preference in male mice. *Pharmacological Research*.

[37] Muller C. L., Anacker A. M. J., Veenstra-VanderWeele J. (2016). The serotonin system in autism spectrum disorder: from biomarker to animal models. *Neuroscience*.

[38] Magne F., Gotteland M., Gauthier L. (2020). The Firmicutes/Bacteroidetes ratio: a relevant marker of gut dysbiosis in obese patients?. *Nutrients*.

[39] Tillisch K., Labus J., Kilpatrick L. (2013). Consumption of fermented milk product with probiotic modulates brain activity. *Gastroenterology*.

[40] McCreight L. J., Bailey C. J., Pearson E. R. (2016). Metformin and the gastrointestinal tract. *Diabetologia*.

[41] Jeong M. Y., Jang H. M., Kim D. H. (2019). High-fat diet causes psychiatric disorders in mice by increasing Proteobacteria population. *Neuroscience Letters*.

[42] Lyte M. (2011). Probiotics function mechanistically as delivery vehicles for neuroactive compounds: microbial endocrinology in the design and use of probiotics. *BioEssays*.

[43] Steenbergen L., Sellaro R., van Hemert S., Bosch J. A., Colzato L. S. (2015). A randomized controlled trial to test the effect of multispecies probiotics on cognitive reactivity to sad mood. *Brain, Behavior, and Immunity*.

[44] Sgritta M., Dooling S. W., Buffington S. A. (2019). Mechanisms underlying microbial-mediated changes in social behavior in mouse models of autism spectrum disorder. *Neuron*.

[45] O'Connor R., van De Wouw M., Moloney G. M. (2021). Strain differences in behaviour and immunity in aged mice: relevance to autism. *Behavioural Brain Research*.

[46] Terzo S., Mulè F., Caldara G. F. (2020). Pistachio consumption alleviates inflammation and improves gut microbiota composition in mice fed a high-fat diet. *International Journal of Molecular Sciences*.

